# Conjoined Twins: The Flip Side

**DOI:** 10.21699/ajcr.v1i1.23

**Published:** 2010-12-01

**Authors:** Tayyab Batool, Jamshed Akhtar

**Affiliations:** Department of Paediatric Surgery, National Institute of Child Health Karachi, Pakistan

**Dear Sir**

The birth of conjoined twins always raises interest among medical community as well as general public [1]. The perspective of parents is often not discussed. In addition, the role of electronic and print media, in creating hype raises many questions [2]. We report a case of conjoined twins (Fig. 1) where many lateral issues influenced the management. 

**Figure:**
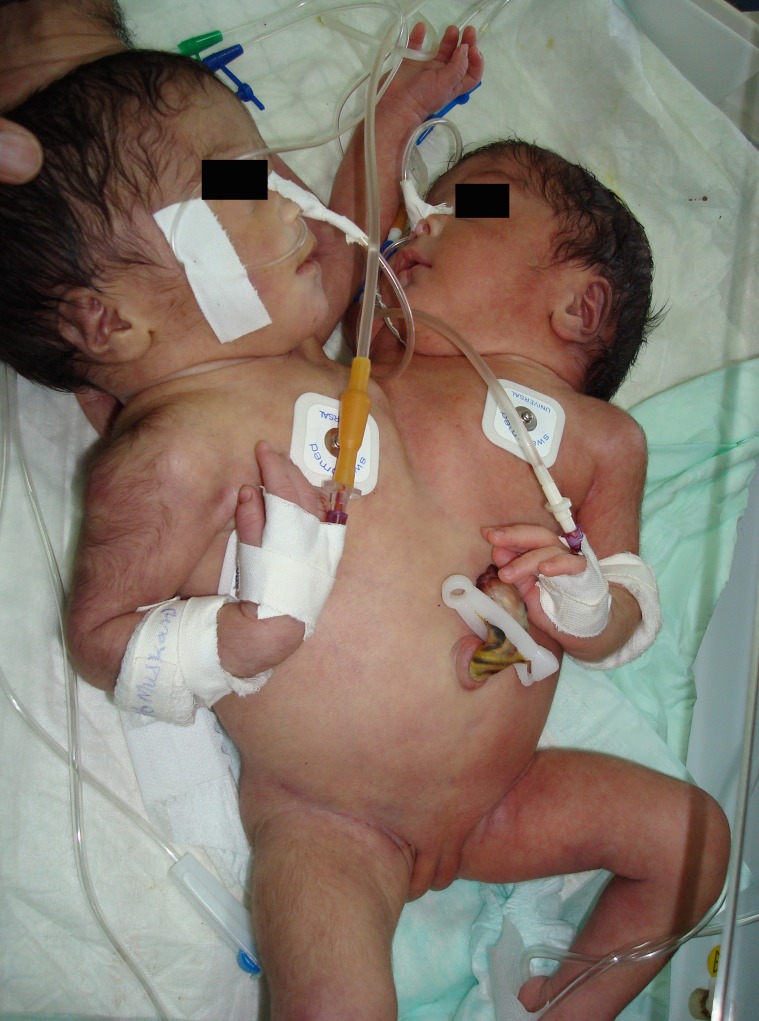
Figure 1: Conjoined twins.

The twins were referred to us for further management after regional pediatric surgical department showed its inability to deal with the case. The family was not counseled and thee transfer was unsupervised. The referring department also made no contact with our team. Babies were brought in, by maternal uncle. Mother did not accompany the newborns, as she was unwell. At arrival babies were not in good condition. One of the twins was having respiratory distress. Immediate resuscitative measures were taken and neonatal care provided. Within hours the decision was made to intubate and mechanically ventilate the pair. However at that time only one ventilator was available, therefore twin 2, who was in critical condition, ventilated. The clinical condition stabilized. Further imaging studies like x-rays, ultrasound and echocardiography were carried out to understand anatomy of fusion and identify associated anomalies.

The family was repeatedly counseled and updated with the progress and future plans discussed. The father of the twins, a labourer, was in Middle East country and not in favour of any treatment. The second major distraction in the management of these twins was print and electronic media. Scores of them were wrangling up and down in the hospital building, around the clock. They were busy interviewing the uncle and kept him under stress. Out of desperation he eventually requested the medical team to either operate or discharge the babies, as he could not wait any further. Although babies started improving clinically but still needed in-hospital care. It was also too premature to decide about separation at this stage, therefore it was decided not to send them home. The family did not agree and left the hospital with the twins.

Many issues surfaced in relation to the case presented. In the absence of social services and patient counselors, decision making is hampered. There is a dire need of such integrated services, especially in paediatric diseases. The role of society in general and media in particular, is very important and has to be re-defined in the background of contemporary practices. Media can educate the society rather than creating hype, by portraying the unusual anomaly. The issues of privacy and confidentiality were violated. Making films without permission, threatening hospital staff, trespassing, violating hospital protocols and forcefully entering into intensive care units, are all illegal but even administration of public sector hospital remained mull in this situation. Incorrect information was telecast without even asking experts in the field of medicine. The treating physicians thus become dejected and disappointed.

In all this debate the one who suffers most is the patient, who is at heart of entire event. In the past surviving conjoined twins were exploited and their anomaly was used to entertain people. It appears that legacy of yesteryears is still persisting in the developing countries like Pakistan, and on this occasion by print and electronic media. It is time to realize that patient and their families with unusual anomalies, have the right to enjoy as normal a life, as possible. Their right to live independently without interference cannot denied.

Treating physicians must understand their responsibilities to patients and their families. Multiple counseling sessions without any interference and influence, in a relax atmosphere must be ensured, so as to make truly informed choices. State too has the responsibility to extend all possible help in such cases. An ethical discourse can change the existing scenario [3]. 

## Footnotes

**Source of Support:** Nil

**Conflict of Interest:** None declared
